# Malaria transmission in Dakar: A two-year survey

**DOI:** 10.1186/1475-2875-7-178

**Published:** 2008-09-16

**Authors:** Frederic Pagès, Gaetan Texier, Bruno Pradines, Libasse Gadiaga, Vanessa Machault, Fanny Jarjaval, Kristell Penhoat, Franck Berger, Jean-François Trape, Christophe Rogier, Cheikh Sokhna

**Affiliations:** 1 Unité d'Entomologie Médicale – Unité de Recherche pour les Maladies Infectieuses et Tropicales Emergentes – Unité Mixte de Recherche 6236, Institut de Médecine Tropicale du Service de Santé des Armées, Bd Charles Livon, Parc du Pharo, 13998 Marseille, France; 2 Unité de Recherche en Biologie et Epidémiologie Parasitaires – Unité de Recherche pour les Maladies Infectieuses et Tropicales Emergentes – Unité Mixte de Recherche 6236, Institut de Médecine Tropicale du Service de Santé des Armées, Marseille, France; 3 Institut de Recherche pour le Développement, Dakar, Sénégal; 4 Département d'Epidémiologie et de Santé Publique Nord, Ecole du Val-de-Grâce, Paris, France

## Abstract

**Background:**

According to entomological studies conducted over the past 30 years, there was low malaria transmission in suburb of Dakar but little evidence of it in the downtown area. However; there was some evidence of local transmission based on reports of malaria among permanent residents. An entomological evaluation of malaria transmission was conducted from May 2005 to October 2006 in two areas of Dakar.

**Methods:**

Mosquitoes were sampled by human landing collection during 34 nights in seven places in Bel-air area (238 person-nights) and during 24 nights in five places in Ouakam area (120 person-nights). Mosquitoes were identified morphologically and by molecular methods. The *Plasmodium falciparum *circumsporozoïte indexes were measured by ELISA, and the entomological inoculation rates (EIR) were calculated for both areas. Molecular assessments of pyrethroid knock down resistance (*Kdr*) and of insensitive acetylcholinesterase resistance were conducted.

**Results:**

From May 2005 to October 2006, 4,117 and 797 *Anopheles gambiae s.l*. respectively were caught in Bel-air and Ouakam. Three members of the complex were present: *Anopheles arabiensis *(> 98%), *Anopheles melas *(< 1%) and *An. gambiae s.s*. molecular form M (< 1%). Infected mosquitoes were caught only during the wintering period between September and November in both places. In 2005 and 2006, annual EIRs were 9,5 and 4, respectively, in Bel-air and 3 and 3, respectively, in Ouakam. The proportion of host-seeking *An. gambiae s.l*. captured indoors were 17% and 51% in Bel air and Ouakam, respectively. Ace 1 mutations were not identified in both members of the *An. gambiae *complex. *Kdr *mutation frequency in *An. arabiensis *was 12% in Bel-air and 9% in Ouakam.

**Conclusion:**

Malaria is transmitted in Dakar downtown area. Infected mosquitoes were caught in two subsequent years during the wintering period in two distant quarters of Dakar. These data agree with clinical data from a Senegalese military Hospital of Dakar (Hospital Principal) where most malaria cases occurred between October and December. It was the first detection of *An. melas *in Dakar.

## Background

In the sixties, the relative seriousness of malaria and the seasonal transmission of *Plasmodium falciparum *by *Anopheles gambiae s.l*. in Dakar were reported in different studies [1-4]. Immunity was acquired quite slowly during the first 20 years of life [5]. Malaria-infected individuals came from rural areas to Dakar and contributed to the start of transmission, which culminated in October-November after the end of the rainy period [6]. In the eighties, an entomological survey proved that malaria transmission occurred in Pikine in the suburb of Dakar and that *Anopheles arabiensis *was the main vector in this area [6]. In the nineties, malaria transmission persisted in Pikine and in the surrounding villages of Dakar, always with *An. arabiensis *as the vector [7]. During the same period, two parasitological and entomological studies conducted in two sanitary districts of Dakar showed that the prevalence of malaria was very low; a few *An. arabiensis *were caught, and none of them were infected by *P. falciparum *[8-10]. Based on these results, many practitioners thought that there was no malaria transmission in Dakar *intra-muros *(*i.e*. in the down-town area) and that the infections occurred in the suburbs or inland. Nevertheless, human malaria cases were reported in autochthonous people who had not been outside Dakar for at least one year, suggesting that malaria was transmitted in Dakar [10]. At the same time, French military doctors were confronted with malaria cases in expatriates, who were visiting a malaria endemic country for the first time, but had never left downtown Dakar. To assess the reality of malaria transmission in Dakar, an entomological evaluation was conducted at the French military bases of Dakar from May 2005 to October 2006, over two winters.

## Materials and methods

### Location

Located at 14°40'20" North, 17°25'22" West (the westernmost point of Africa), Dakar, the capital city of Senegal, has 1,030,594 inhabitants and covers the major part of the Cap Vert Peninsula. The altitude does not exceed 104 m. The population of the Dakar area is estimated to be 2.45 million people, representing 20% of the Senegalese population. The estimated population density is 12,233 inhabitants/km^2^.

**Figure 1:**
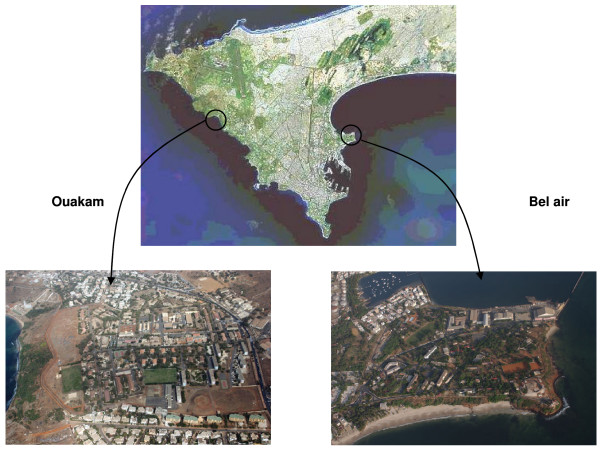
Localization and surroundings of the French military camps in Dakar.

The study was conducted in two districts of Dakar: Bel-air in the east of the city and Ouakam in the west (Figure 1). Bel-air is a residential district with luxuriant vegetation and with many market gardens and water wells along a railway, which crosses the area. The French military camp is bordered by the sea on three sides and by the railway on the other side. Ouakam is also a residential district with individual and collective houses. The French military camp of Ouakam is bordered by the sea on the west side and by houses on the other sides. It is a dry area with little vegetation. Between the camp and the sea lie a market gardens area and two water wells. The waste water of the French camp flows into a network that irrigates all the market gardens.

### Climate

The Cap Vert Peninsula is located in the Atlantic Sudan zone. Two distinct seasons exist: a hot and wet season from June to November (maximum average temperature 28.2°C in October) and a cool and dry season from December to May (minimum average temperature 20.4°C in February). The first rains generally occur at the end of June or the beginning of July, and the last occur at the beginning of October. In 2005 and 2006 (the period covered by the study), the average rainfall was 525 and 350 mm, respectively.

### Field mosquito processing

Sampling by human landing of malaria vectors was carried out both indoors and outdoors. Collectors gave prior informed consent and received anti-malaria prophylaxis and yellow fever immunization. Collectors were organized in teams of two for each collection point. Replacement of workers within a team was done every two hours from 7:00 PM to 7:00 AM. Teams of collectors were rotated among the collection points on different collection nights to minimize sampling bias. Landing catches were performed at seven points (two places indoors and five outdoors) in two periods, from May 2005 to May 2006 and from September to October 2006, during 34 nights in Bel-air (i.e. 238 person-nights). In Ouakam, human landing collections were performed at five points (two indoor and three outdoor locations) for two periods, from July 2005 to May 2006 and from September to October 2006, during 24 nights (i.e. 120 person-nights).

Mosquitoes were recorded by the location and hours of capture. They were sorted by genera, and anopheline mosquitoes were identified morphologically following the Gillies and Coetzee keys and by software from Hervy *et al *[11,12]. Culicinae were identified morphologically following the Edwards keys [13]. All mosquitoes were stored individually in numbered vials with desiccant and preserved at -20°C until processing at the Medical Entomology Unit of the Institute for Tropical Medicine (IMTSSA), Marseille (France).

### Laboratory mosquito processing

Heads and thoraces of anopheline females were tested by enzyme-linked immunosorbent assay (ELISA) for *P. falciparum *circumsporozoite protein (CSP)[14]. All females belonging to the *A. gambiae *complex caught during the dry season, a random sample of females caught during the rainy season, together with all CSP-positive anopheline, were identified by polymerase chain reaction (PCR) at the species and molecular forms levels [15]. Molecular characterizations of the *Kdr *and *Ace1 *mutations were carried out on these mosquitoes as previously described [16,17].

### Data analysis

The human biting rate (HBR) was expressed as the number of female anopheline bites per human per night. The CSP index was calculated as the proportion of mosquitoes found to be positive for CSP. The entomological inoculation rate (EIR) was calculated as the product of the HBR and the CSP index of mosquitoes collected on humans. The *An. gambiae s.l*. biting activity is sufficient for transmission only during the end of the rainy period; the EIR calculated for this period will be considered as the annual EIR. Conformity of *Kdr *and *Ace1 *frequencies with Hardy-Weinberg expectations was tested using a Pearson chi-square test considered significant when P < 0.05. Endophagic rates were compared using a chi-square test.

### Weather data

Rainfall data were graciously provided by the National Weather Agency.

## Results

### Mosquito collection

A total of 69,082 mosquitoes were caught (76.4% *Culex quinquefasciatus*, 13.9% *Culex tritaeniorynchus*, 7.1% *An. gambiae s.l.*, 2.2% *Aedes aegypti*; Tables 1 and 2).

**Table 1:** Mosquitoes collected in the French military camps of Dakar from May 2005 to May 2006: distribution by species, camps, periods and place of capture (indoor or outdoor).

	From May 2005 to May 2006				
	**Bel-air**		**Ouakam**		TOTAL
	Indoor	Outdoor	Indoor	Outdoor	

*Anopheles gambiae s.l*.	346	2703	242	347	3638
*Anopheles pharoensis*	2	17	0	1	20
*C. quinquefasciatus*	12024	26407	1480	3568	43479
*C. tritaeniorynchus*	419	5230	71	1585	7305
*Aedes aegypti*	113	964	26	256	1359
*Aedes metallicus*	2	81	4	82	169
*Aedes vitattus*		7			7
*Mansonia sp*		4			4
*Aedeomyia sp*				2	2

**Table 2:** Mosquitoes collected in the French military camps of Dakar from September to October 2006: distribution by species, camps, periods and place of capture (indoor or outdoor).

	From September to October 2006				
	**Bel-air**		**Ouakam**		TOTAL
	Indoor	Outdoor	Indoor	Outdoor	

*Anopheles gambiae s.l*.	78	990	23	185	1276
*Anopheles pharoensis*	2	4			6
*C. quinquefasciatus*	858	7713	252	475	9298
*C. tritaeniorynchus*	43	2100	3	159	2305
*Aedes aegypti*	2	156	8	34	200
*Aedes metallicus*					0
*Aedes vitattus*		3			3
*Mansonia sp*		11			11
*Aedeomyia sp*		0			0

### Biting rates and biting behaviour of *An. gambiae s.l*

**Figure 2:**
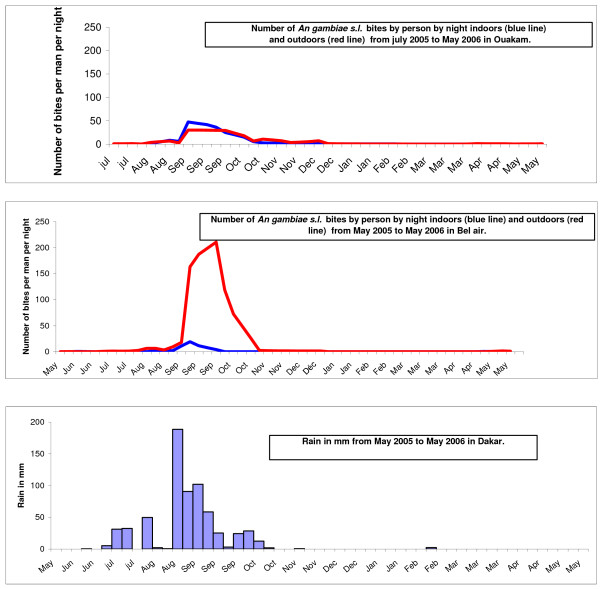
Outdoor and indoor aggressiveness of *An. gambiae s.l*. in Ouakam and Bel air from May 2005 to May 2006, and rainfall.

**Figure 3:**
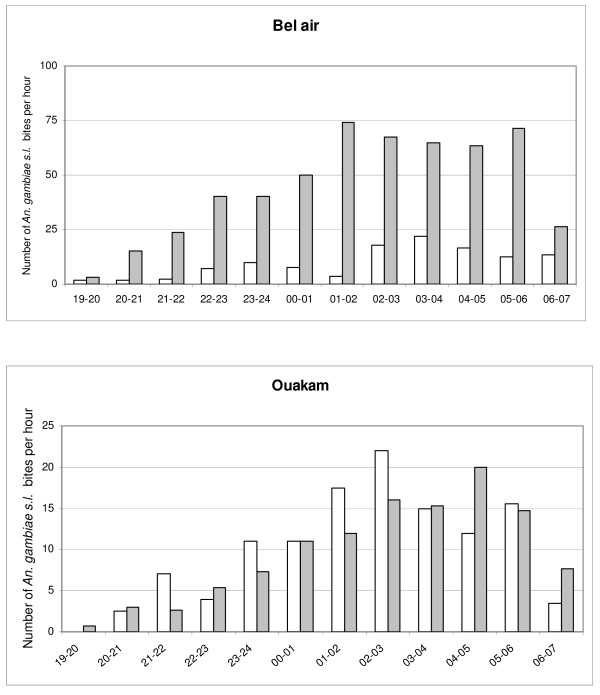
Distribution by hours of *An. gambiae s.l*. bites indoors (white bars) and outdoors (gray bars) in Dakar, May 2005 to May 2006.

The biting activity at the two sites from May 2005 to May 2006 is shown in Figure 2. *An. gambiae s.l*. was present throughout the year in Dakar, but most of the specimens (98%) were caught between July and December. The peak of biting occurred in September and October, at the end of the winter period (rainy season): 67% and 87% of mosquitoes were caught during these two months in Ouakam and Bel-air, respectively. During this period in 2005, in Bel-air, the average biting rate for *An. gambiae s.l*. was 112 bites per person per night, with a peak of 181 bites per person per night; in Ouakam, the average biting rate for *An. gambiae s.l*. was 19.7 bites per person per night, with a peak of 37.2 bites per person per night. During September-October in 2006, the average rates in Bel-air and Ouakam were 19.1 and 10.4 *An. gambiae s.l*. bites per person per night, respectively. The distribution of *An. gambiae s.l*. bites by hour is shown in Figure 3. Indoors over 70% of biting occurred between 1:00 a.m. and 6:00 a.m. in Ouakam and between 2:00 a.m. and 7:00 a.m. in Bel-air; outdoors, over 70% of biting occurred between 1:00 a.m. and 6:00 a.m. in both locations.

The average number per catching point of host-seeking *An. gambiae s.l*. caught indoor and outdoor were 121 and 115, respectively in Ouakam and 115 and 540, respectively in Bel air. The proportion of host-seeking *An. gambiae s.l*. captured indoors were 51% and 17% in Ouakam and Bel air, respectively. (P < 0.0001, RR = 2.92 [2.37; 3.59], indicating that this species was more endophagic in Ouakam.

### CSP and EIR

In Bel-air, 3,049 *An. gambiae s.l*. collected by human landing catches were processed by ELISA for *P. falciparum *antigen detection in 2005, and 1,084 were processed in 2006. Assuming that malaria transmission could occurred only in the end of the rainy season, CSP index has been calculated only from the end of September to December. From May 2005 to the middle of September 2005, none of the 1,546 mosquitoes were positive for CSP. From the end of September to December, seven specimens among 1,503 were CSP positive. The CSP index was 0.46% (CI95% = 0.19–0.96). From December 2005 to May 2006, none of the 16 mosquitoes were positive for CSP. In September and October 2006, three specimens among 1,068 were positive. The CSP index was 0.28% (CI95% = 0.06–0.82).

Infected mosquitoes were identified only between the end of the rainy season. In 2005 and 2006, annual EIRs were 9.5 and 4 infective bites for a person without protection, respectively.

In Ouakam, 589 *An. gambiae s.l*. collected by human landing catch were processed by ELISA for *P. falciparum *antigen detection in 2005, and 217 were processed in 2006. From July to the middle of September 2005, none of the 279 mosquitoes were positive. From the end of September to December, two specimens among 310 were positive. The CSP index was 0.64% (CI95% = 0.08–2.30). From December 2005 to May 2006, none of the 9 mosquitoes were positive. In September and October 2006, two specimens among 1,068 were positive. The CSP index was 0.96% (CI95% = 0.12–3.40). In 2005 and 2006, annual EIRs were 3 and 3 infective bites for a person without protection, respectively.

### Molecular identification of *An. gambiae s.l*

Using rDNA-PCR, all specimens of *An. gambiae s.l*. captured before September 2005 (373 from Bel-air, and 82 from Ouakam), a random sample of the mosquitoes caught from September to December 2005 (346 from Bel-air and 216 from Ouakam), a random sample of the mosquitoes caught in September and October 2006 (137 from Bel-air and 134 from Ouakam), and all CSP-positive specimens were identified at the species level. In Bel-air, three members of the complex were present in 2005: *An. arabiensis *(97.8%), *Anopheles melas *(2.0%) and *An. gambiae s.s*. molecular form M (0.2%). In 2006, only *An. arabiensis *(98.5%) and *An. gambiae s.s*. molecular form M (1.5%) were present. In Ouakam, and only *An. arabiensis *was present in 2005. In 2006, one specimen of *An. melas *was caught. Emerging adults and all CSP-positive specimens were *An. arabiensis*.

### *Kdr-w *and Ace1 mutation frequencies in *An. gambiae s.l*

All CSP-positive mosquitoes per site and a random sample of 100 PCR-identified mosquitoes were tested for the *Kd-wr *and *Ace1 *mutations. Ace 1 mutations were not identified in any members of the *An. gambiae *complex either in Ouakam or in Bel-air. The *Kdr-w *mutation frequency in *An. arabiensis *was 12% in Bel-air and 9% in Ouakam (not a significant statistical difference). The genotypic frequencies are shown in Table 3. The populations were not at Hardy-Weinberg equilibrium at either site for kdr (P < 0.0001).

**Table 3:** Genotypic frequencies for *Kdr *loci of two samples of *An. arabiensis *collected in the French military camps of Bel air and Ouakam from May 2005 to May 2006.

	Bel-air	Ouakam
RR	11 (0,10)	5 (0,05)
RS	4 (0,04)	9 (0,09)
SS	94 (0,86)	90 (0,86)
Total	109	104

## Discussion

Malaria transmission is a reality in Dakar. *P. falciparum*-infected Anopheles have been caught during two rainy periods consecutively in two quarters of the town. All infected *An. gambiae s.l*. were caught only during the rainy period. The seasonal transmission occurs in the end of the rainy season from September to November. In August 2005, very heavy rains (278 mm of water in six days) flooded many parts of the city. Many potential breeding sites were created, and the *An gambiae s.l*. aggressiveness was high, with a peak of almost 200 bites per person per night in September. This abnormal situation in Dakar, due to an exceptional climatic event, could have led to an exceptional malaria transmission. However, the catch of infected Anopheles during the 2006 winter proved that malaria transmission is not exceptional and occurred each year during this period. These data agree with clinical data from a Senegalese military Hospital of Dakar (Hospital Principal), where most malaria cases occur between October and December.

Only specimens of *An. arabiensis *were infected. *Anopheles arabiensis *is the main member of the gambiae complex in Dakar and, according to its abundance and to the results of this study, is the main malaria vector. The presence of *An. melas *was detected in Dakar for the first time in the present study. Nevertheless, this vector was not found to be infected with *P. falciparum*. Until now, *An. melas *had been reported only in the mangrove swamps of the Delta's Saloum (south Senegal) and in the Senegal River delta (north Senegal) [18,19]. In addition, neither *Anopheles pharoensis *nor *An. gambiae *molecular form M caught in Dakar were infected with *P. falciparum*. The aggressiveness of *An. arabiensis *was higher in Bel-air than in Ouakam, but EIRs were very high in the two quarters. *An. arabiensis *is more endophagic in Ouakam. This behavior allows for an easier access to a human blood meal that may explain the similar EIRs observed for the two quarters.

The seasonal character of transmission allows us to calculate mean annual EIR for the two years of study. In Bel-air, the mean annual EIR fell from 9.5 in 2005 to 4 in 2006. In Ouakam, a similar mean annual EIR of 3 was estimated for 2005 and 2006. Our observations are consistent with the results of a meta-analysis of studies of malaria transmission in sub-Saharan Africa, which found a mean annual EIR of 7.1 in the city centers, with more than two-thirds of the studies reporting EIRs < 4/year [20]. In 1996, the number of infective bites per person was estimated to 0.05/year for the central area of Dakar, i.e., one infective bite every 20 years [10]. This work was conducted on a very large area with 13 study sites but with only two catching points per site and human landing collections performed once every month. Ten years later, the risk of malaria transmission seemed to be 60 to 80 times higher. The peak of *An. gambiae s.l*. aggressiveness lasts for a little longer than one month (Figure 3). With a monthly rhythm of capture, the previous study could have missed this peak. Nevertheless, a modification of the entomological situation over ten years cannot be excluded. There is some evidence that anopheline species may be adapting to urban ecosystems. Adaptation of *An. gambiae s.s*. to urban aquatic habitats, such as water-filled domestic containers, has been observed in Accra, Ghana [21]. In addition, adaptations of the anopheline vectors to new breeding sites (tree holes, polluted water) are reported from many urban areas in Africa [22-24]. The impact of urbanization on the composition of the vector system and malaria transmission dynamics has been highlighted in many studies [25,26]. Urban farming provides ample aquatic habitats for mosquitoes, which are responsible for the persistence of anopheline populations in many African towns [27-29]. In Ouakam and Bel-air, as in other places in Dakar, market gardens are present with or without water wells (cement wells or traditional wells called "ceanes"). The urban area of Dakar contains more than 5,000 market-garden wells, which provide permanent sites for mosquito larvae, in particular *An. Arabiensis *[30]. The increase of the *An. arabiensis *population size during the 2005 winter is an argument to highlight the major role of temporary pools in malaria transmission in Dakar. Further specific study is necessary to understand the impact wells and urban farming on anopheline density and malaria transmission.

This situation questions the origins of these anopheline populations. Are there autochthonous populations from Dakar growing during the rainy season, or are they coming in from other areas of Senegal? Populations of *An. arabiensis *in West Africa are considered to be continuous throughout the year, with many individuals surviving through the dry season, perhaps in a physiologically altered state rather than through extinction or a severe bottleneck during the dry season, followed by re-colonization by a few individual survivors or immigrants in the subsequent rainy season [31]. In Barkedji, Senegal, Simard *et al *did not detect any difference in measures of genetic diversity and linkage disequilibrium between the dry and rainy seasons [32]. They concluded that, despite extreme minima in local density, malaria transmission in this area was due to autochthonous population of *An. arabiensis*. They also found a low differentiation between two populations, which were 250 Km apart, suggesting extensive gene flow across this distance. These results suggest that *An. arabiensis *maintains a large permanent deme over a large area. The situation in Dakar seems similar to that in Barkedji: a long dry season where no *An. arabiensis *(or only a few) are caught [33]. Further genetic studies will be necessary to confirm the hypothesis that malaria transmission is due to an autochthonous *An. arabiensis *population, and to assess the gene flow between the urban and rural populations of Senegalese *An. arabiensis*.

Pyrethroids are the main insecticide used in malaria vector control, including indoor residual spraying and impregnated materials (bednets, curtains, plastic sheeting). Pyrethroids have the advantage of acting very rapidly as insecticides, with both knockdown and lethal effects at dosages under the threshold of mammalian toxicity [34]. Since 1970s, pyrethroids have been extensively used in urban areas and for agricultural purpose in rural areas. Detected in the 1990s, knock-down resistance (*Kdr*) to pyrethroids and DDT of *An. gambiae s.l *is an increasing problem. Two mutations at the same locus in the voltage-gated sodium channel are known to confer knock-down resistance to a wide range of pyrethroids and DDT [35-37]. These mutations were previously described in west and east Africa (*Kdr-w *and *Kdr-e*) in *An. gambiae s.s*. as well in *An. arabiensis *[38-41]. There are few studies of *An. arabiensis *insecticide susceptibility in the area of Dakar. In 1987, bioassays were conducted in two places in the suburbs of Pikine and of Thiaroye [42], where in vivo resistance to DDT was observed. The agricultural use of DDT in market gardens was incriminated. In 1999, a normal susceptibility of *An. arabiensis *was found in Dakar [43]. Our data show a *Kdr-w *frequency of 0.12 in Bel-air and 0.09 in Ouakam. The populations were not at Hardy-Weinberg equilibrium for *Kdr *in either site (P < 0.0001). This disequilibrium could be due to local selection pressure by the agricultural use of pyrethroids in market gardens.

The *Kdr *mutation has been shown to be closely associated with DDT and pyrethroid resistance in several *An. gambiae *populations (particularly the molecular S form) [35-37]. However, the role of *Kdr *in conferring resistance in *An. arabiensis *remains unclear [44,45] As a result, insecticide susceptibility tests should be carried out to assess physiological resistance levels in *An. arabiensis *in Dakar. However, this is the first report of the *Kdr-w *mutation in *An. arabiensis *in Dakar. Resistance to carbamate and organophosphate insecticides is also widespread in West Africa. The presence of an insensitive acetylcholinesterase in populations of *An. gambiae s.s*. of both forms was revealed by biochemical assays. The ACE-1-R mutation has also been detected in the two molecular forms of *An. gambiae s.s *in many West African countries [17,46]. This mutation has not been detected in *An. arabiensis *at present. Our results are consistent with these data.

In terms of effective vector control at the military camps and in town, choice of insecticide should depend on the results of susceptibility tests on *An. arabiensis*.

Malaria prevalence is very low in Dakar and its urban periphery [7,8,10]. Nevertheless, cerebral malaria is the first etiology of neuromeningeal diseases in Dakar [47]. Extensive genetic diversity was observed in *P. falciparum *isolates collected in Dakar [48-50]. Significant linkage disequilibrium was observed with microsatellite loci in urban parasites. Two non-exclusive hypothesis could explain the situation in Dakar: (i) a global non-panmictic structure of Dakar malaria population due to a high predominance of selfing; (ii) a structuration in subpopulations of several malaria foci in Dakar. The entomological findings of the present study are consistent with seasonal transmission leading to an increase of cases in the October end of the rainy season. There is a concern about the origin of *Plasmodium falciparum *infections observed in Dakar during the rainy season. Are they due to autochthonous parasites transmitted during the winter or to parasites imported from the suburbs by commuters, as suggested by Vercruysse? [6] In Senegalese people employed by the French army, the first malaria cases are diagnosed in commuters at the beginning of the rainy season, and cases in residents occur later in the season. The movement of populations from the suburbs or rural areas to Dakar must be considered to understand malaria epidemiology in Dakar [51,52].

Urban malaria is considered to be an emerging problem in Africa. In 2003, 39% of Africa's people lived in urban settings; by 2030, 54% of Africans are expected to do so [53]. With the increase of people living in urban dwellings, it's important to develop and validate new approaches for rapid appraisal of malaria risk. The rapid urban malaria appraisal (RUMA) methodology has been develop to provide a cost effective tool to conduct assessment of the malaria situation in urban sub-Saharan Africa and to improve the understanding of urban malaria [54]. The only entomological point required is in this evaluation is the mapping of breeding sites. Considered very time-consuming, this task has been done only in Ouagadougou [27].

The results of this study confirm that the vector complex situation in African towns is always changing, with the description of *An melas *and *An gambiae s.s*. in Dakar. Entomological studies are long, difficult and require time and special expertise but they are indispensable to understand the dynamics of malaria transmission in urban settlements and to monitor the increase of the insecticide resistance in urban mosquitoes. The study showed a difference in host finding behaviour between the two quarters: *An. arabiensis *are more endophagic in Ouakam than in Bel air. This difference of behaviour has an impact on the malaria transmission level. With a lower biting activity during the transmission season, the Annual EIR in Ouakam remains as high than in Bel air. Indoor vector control measures will probably not have the same impact in the two quarters. The misuse of impregnated bed nets during this period is probably riskier in Ouakam.

Malaria is transmitted in Dakar. This seasonal transmission occurs only during the two last months of the rainy season. The transmission level could be very high. Further studies have to be conducted in other parts of Dakar to assess the risk of transmission, to understand the role of permanent and temporary pools, the impact of urban farming and to discover the origin of the Dakar anopheline populations.

## Competing interests

The authors declare that they have no competing interests.

## Authors' contributions

FP was responsible for the study design, supervision of data collection, analysis, interpretation and production of the final manuscript and revisions. GT contributed to the supervision of data collection, the data analysis, interpretation and production of final manuscript. BP contributed to the supervision of data collection, to the data analysis, interpretation and production of final manuscript. LG contributed to the supervision of data collection, to the data analysis, interpretation and production of final manuscript. VM contributed to the data analysis and to the preparation of the final manuscript. FJ contributed to the supervision of data collection, to the data analysis. KP contributed to the supervision of data collection, to the data analysis. FB contributed to the supervision of data collection, to the data analysis. JFT was contributed to overall scientific management, analysis, interpretation and preparation of the final manuscript and revisions. CR was contributed to overall scientific management, analysis, interpretation and preparation of the final manuscript and revisions. CS was responsible for overall scientific management, analysis, interpretation and preparation of the final manuscript and revisions. All authors read and approved the final manuscript.
